# Potential Role of 3-Dimensional Printed Vascular Models in Maintenance Hemodialysis Care

**DOI:** 10.1016/j.xkme.2021.07.006

**Published:** 2021-09-24

**Authors:** Yi Li, Aliza Anwar Memon, Ayaz Aghayev, Kanmani Kabilan, Tuan Luu, Li-Li Hsiao, Sijie Zheng, Matthew S. Chin, Codi Ghargouzloo, Andrew Siedlecki

**Affiliations:** 1 Brigham and Women’s Hospital, Renal Division, Department of Medicine, Boston, Massachusetts; 2 Brigham and Women’s Hospital, Department of Radiology, Boston, Massachusetts; 3 Kaiser Permanente, Oakland, California; 4 Geisinger Health System, Wilkes Barre, Pennsylvania; 5 Imaginostics Inc, Cambridge, Massachusetts

**Keywords:** Hemodialysis, vascular access, infiltration, hemodialysis access cannulation, three dimensional printing

## Abstract

Infiltration of a surgically placed hemodialysis vascular access is recognized as a major contributor to the high health care costs associated with dialysis-dependent patients. Three-dimensional (3D) modeling is a critical tool for proceduralists in preparation for surgical interventions. No such modeling is currently available for dialysis specialists to avoid the common complication of vascular access infiltration. Ferumoxytol-enhanced magnetic resonance angiography was used to generate 3D image data that could render a 3D resin-based model of a vascular access without exposing the patient to iodinated or gadolinium-based radiologic contrast. The technique required an abbreviated magnetic resonance angiography procedure interfaced with a 3D printer workstation. An interventional radiology suite was not required. In the described case, the brachial artery was clearly delineated from a cephalic vein to basilic vein bypass with a 3D spatial resolution of 1 mm. In conclusion, we demonstrate that this new technology pathway can provide preprocedural guidance that has the potential to significantly reduce the morbidity and cost associated with vascular access infiltration.

## Introduction

To ensure adequate kidney replacement therapy, patent surgically placed hemodialysis vascular access is critical. The success of maintaining a functional vascular access is central to the Fistula First initiative^1^ but is limited by various vascular access complications, including infiltration, stenosis, and thrombosis. The most commonly encountered complication in the outpatient hemodialysis environment is access infiltration.^2^ Twenty to thirty percent of infiltrations have been associated with subsequent fistula thrombosis. This complication rate leads to expensive corrective interventions, including thrombectomy, angioplasty, temporary dialysis catheter placement, and access salvage via vascular surgery. In total, 50% of the annual cost of dialysis care can be directly attributed to vascular access infiltration.^3^ Despite the recognized health care cost associated with vascular access infiltration, dialysis specialists are offered little preprocedural guidance tailored to the often complex vascular anatomy and individual access.

Radiologic imaging plays a pivotal role in the pre- and postoperative assessment of the vascular access. Modalities such as Doppler ultrasonography, digital subtraction angiography, and multidimensional computed tomography angiography are available but limited in their application due either to restriction of Cartesian-plane imaging^4^ or potential side effects.^5^, ^6^, ^7^ Ferumoxytol-enhanced magnetic resonance angiography (FeMRA) provides accurate 3-dimensional (3D) imaging of vascular structures that can be readily manipulated in the x-y-z axes while simultaneously offering treatment for iron deficiency anemia.^8^^,^^9^ It provides a practical solution for the generation of preprocedural modeling to reduce patient morbidity and the health care cost associated with vascular access infiltration.

## Case Report

**Figure 1:**
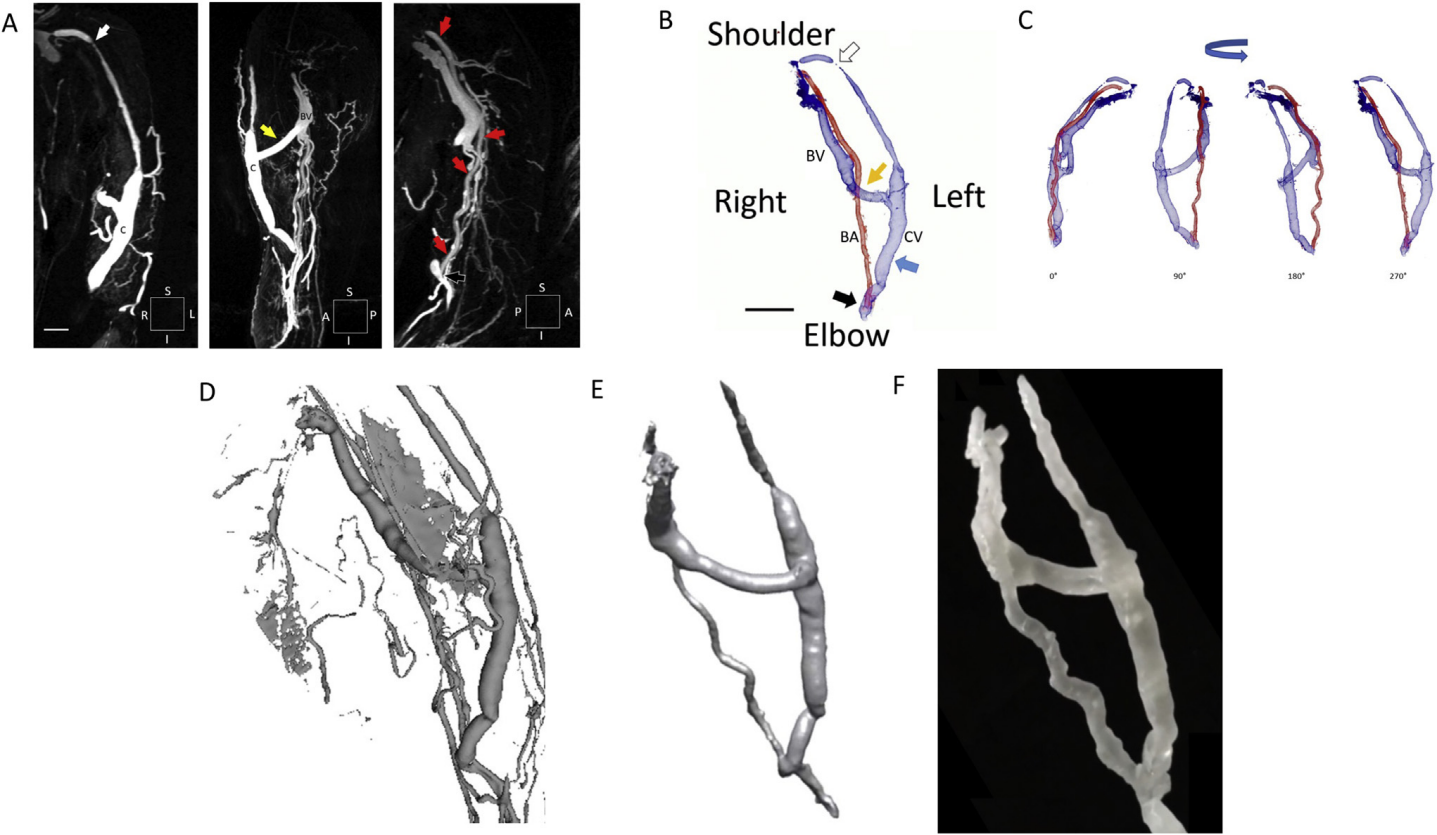
Ferumoxytol-enhanced magnetic resonance angiography of a surgically placed brachial artery (BA) to cephalic vein (CV) fistula revised with a CV to basilic vein (BV) bypass. (A) Left CV (C) and stenosis (white arrow), CV (C) to BV (A) bypass graft (yellow arrow) due to CV stenosis, BA (red arrows) proximity to BV (A) (black arrow) (white arrow) (scale bar, 2 cm). (B) DICOM-based 3D image reconstruction (scale bar, 5 cm) of a hemodialysis vascular access using ferumoxytol-enhanced magnetic resonance angiography. Left BA (red) to CV (blue arrow) underwent CV to BV bypass graft (yellow arrow) due to CV stenosis (white arrow). (C) Multiple views of DICOM-based 3D reconstruction. (D) STL-based 3D reconstruction. (E) Distilled STL-based data reconstruction. (F) 3D print of resin-cured access model. Abbreviations: A, anterior; I, inferior; P, posterior; S, superior.

Using a clinical protocol overseen by the local institutional review board, FeMRA was performed by infusing 3 mg/kg of ferumoxytol over a 10 minute period. It was performed in a patient requiring maintenance hemodialysis with a residual kidney function of 7 mL/min/1.73 m^2^ who successfully underwent cephalic to basilic vein bypass using a polytetrafluoroethylene graft due to cephalic vein stenosis 4 months before FeMRA. Image acquisition was initiated after infusion (Fig 1A). Standard image coordinate system-based data acquisition was recorded in Digital Imaging and Communications in Medicine (DICOM) files. Cephalic vein stenosis, cephalic vein bypass, and the brachial artery could be visualized, but the demarcation between the basilic vein and the brachial artery was less apparent. The initial images were then used to generate a high-resolution (1 mm^3^) 3D image reconstruction of both cutaneous and deep vascular structures with a more clear delineation of venous and arterial blood vessels (Fig 1B). 3D reconstructed MRA demonstrated the proximity of the brachial artery to the basilic vein (Fig 1B and C; Movie S1). The position of the newly dilated basilic vein was <0.5 cm from the brachial artery compared to 4-8 cm of linear distance along much of the cephalic vein.

**Figure 2:**
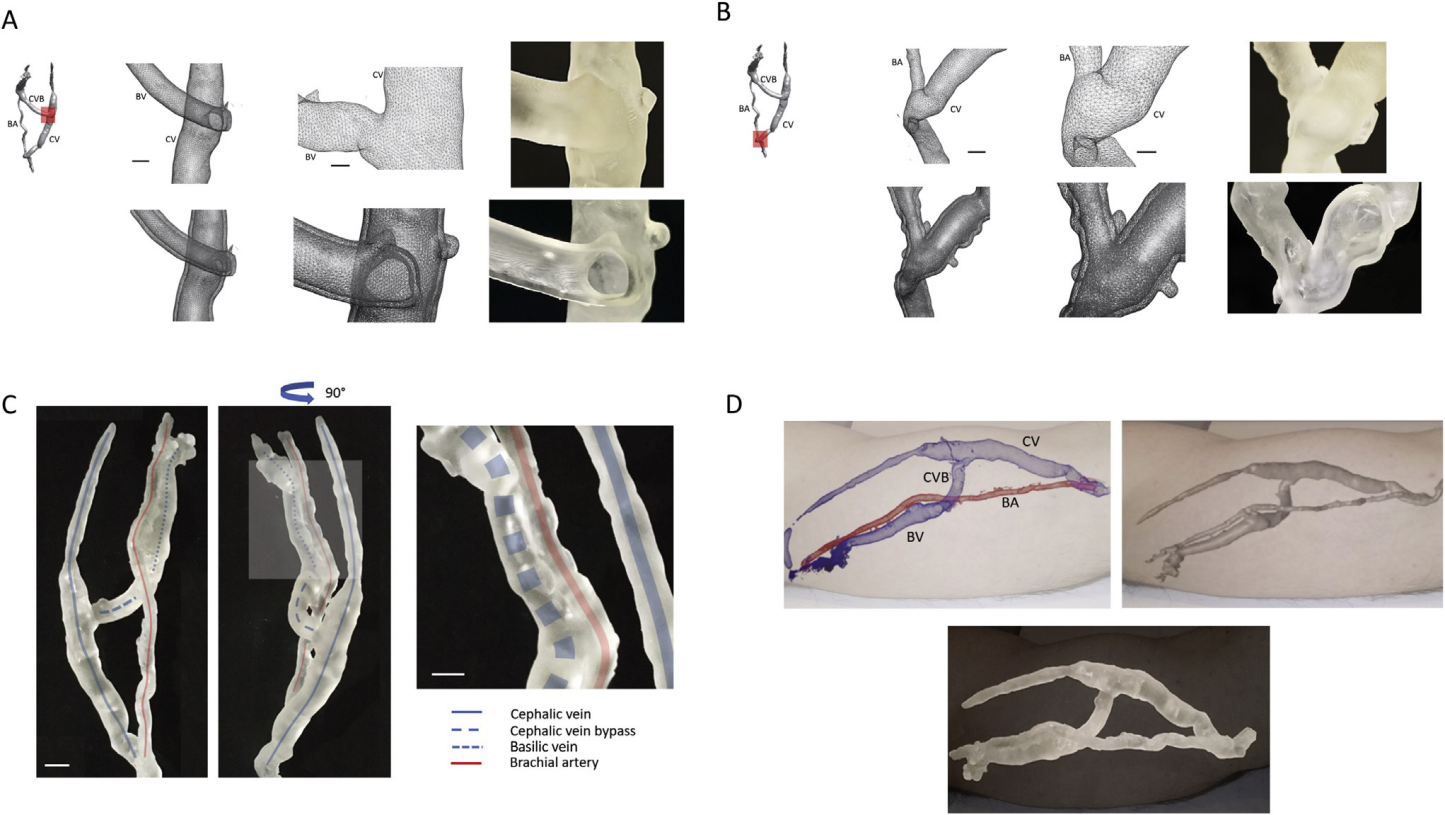
Unique characterization of a surgically placed brachial artery (BA) to cephalic vein (CV) fistula revised with a CV to basilic vein (BV) bypass using 3D printing techniques. (A) Left CV site displaying triangular curvature reconstruction of the CV to BV bypass (red box) with corresponding STL-formatted vascular access model of the cephalic vein bypass (top row, second panel) (scale bar, 1 cm). High-resolution solid slab section modeling using triangular curvature reconstruction, magnified view (top row, third panel) (scale bar, 0.5 cm) and 3D print of resin-cured access model (top row, fourth panel). Conversion of solid slab model to hollow section model (bottom row, first panel), further delineating the cephalic vein bypass, with magnified view (bottom row, second panel) and 3D print of resin-cured access model (bottom row, third panel). (B) Digital triangular curvature reconstruction highlighting the BA to CV anastomosis (first panel) (red box). High-resolution solid slab section model delineating the anastomosis of the BA to the CV (top row, second panel) (scale bar, 1 cm), magnified view (top row, third panel) (scale bar, 0.5 cm) and 3D print of resin-cured access model (top row, fourth panel). Conversion of solid slab model to hollow section model further delineating intraluminal calibre of the BA and CV (bottom row, first panel), magnified view (bottom row, second panel) and 3D print of resin-cured access model (bottom row, third panel). Cured-resin model of a hemodialysis access. (C) Free-standing model showing BA (red line), CV (blue line), BV (blue dotted line) and cephalic vein bypass (CVB, blue hashed line) (first panel). Rotated model showing proximity of BA along the length of the BV (second panel), magnified view of overlapping BA (red line) and BV (blue shading) (third panel). (D) Dialysis access imaging in relation to visualized anatomy using DICOM format (first panel), STL format (second panel), and 3D printed model (third panel).

DICOM data files were reorganized for linked Cartesian-plane stacking using Standard Tessellation Language (STL) formatting in preparation for 3D printing applications. STL files were subsequently formatted for 3D model rendering using open-source software (3D Slicer v 4.11.0) (Fig 1D and E).^10^ Data was further distilled to clarify the arterial and venous structures using additional open-source software (Omri Rips v1.00). The computed geographic design was delivered to a 3D printer (Formlabs Inc) using a standard scalable vector graphic format (Fig. 1F). High-resolution slab section modeling was made available using triangular curvature reconstruction techniques (Fig 2A-C; Movies S2-S5). Subsequently, a computational algorithm was applied to transform the slab-fill matrix to a hollow-lumen matrix rendering a model that could demonstrate intraluminal diameters proportional to the composite vascular landscape of the vascular access. Because the radiologic contrast agent used was a blood pool agent, large data samples could be acquired from the interrogated anatomical region providing a 3D intraluminal map with 1 mm^3^ resolution in 1:1.49 scale, thus closely reflecting the unique intravascular volume of the patient’s hemodialysis access. Liquid ultraviolet light-curable resin-based model expansion was completed within 120 minutes (Movie S6). The 3D slab model in this example demonstrated the proximity of the brachial artery to the basilic vein (Fig 2C, Movie S7). The maintained proportionality of the design was demonstrated when comparing 3D MRA reconstruction, 3D STL reconstruction, and the 3D cured-resin model to overlying cutaneous left arm landmarks (Fig 2D).

The time required for the complete process of model manufacturing was 4 hours and 30 minutes including FeMRA image acquisition (30 minutes), data conversion (120 minutes), and model printing (120 minutes). This accelerated timeframe was made possible by the routine nature of the clinical image acquisition and standard 3D printing techniques. After model construction, the cured-resin composition allowed for model delivery to the patient’s outpatient dialysis center using standard shipping methods (Fig S1). A brief video (Movie S8) was provided to the dialysis center to demonstrate to the hemodialysis specialist the subcutaneous and deep features of the patient’s vascular access. Once the model was printed, it could be kept on hand in the hemodialysis therapy area for the purpose of staff training and reassessment.

The estimated impact of 3D-printed vascular access models on health care costs is complex. Invasive diagnostic angiography (ie, fistulogram) is approximately 3.82-fold more expensive than an MRA of the upper extremity.^11^ However, when the anticipated cost of 3D anatomical modeling is accounted for, invasive diagnostic angiography is 3.61-fold more expensive than MRA of the upper extremity. Additional costs of invasive diagnostic angiography not included in this calculation involve requirements for anesthesia. The cost associated with hospital readmission rates for patients with 3D-printed vascular access models compared to standard of care cannot be estimated at this time.

## Discussion

Until now, radiologic imaging has not been used to construct 3D models of the vascular access for the benefit of dialysis specialists. Thus far, FeMRA has been used selectively to assess abdominal and thoracic vasculature. The upper arm has been imaged with FeMRA in the past in preparation for vascular surgery, but the utility of imaging the vascular access for outpatient hemodialysis centers was not realized at the time, due in part to the nascent phase of medical modeling.^12^ The routine nature of the workflow now has the potential to be used for a broad patient population that may benefit from 3D medical modeling.

In this study, we use an imaging technique that has become commonly used in patients with compromised vascular access function. We now provide a practical tool that can immediately enhance the techniques of a dialysis specialist and facilitate vascular access care. In particular, access revisions with complicated anatomy can now have models rendered within days following the vascular surgery to further guide the anatomical landmark assessments dialysis specialists currently use in preparation for cannulation. The model is unique to each patient, emphasizing to specialists in training that cannulation techniques must be personalized to each patient.

Limitations of this study center on the side effect profile of ferumoxytol as an intravenous iron formulation. Ferumoxytol should not be used in patients with a history of anaphylaxis associated with intravenous iron infusion. Further, extreme caution should be taken when considering this technique in patients with diagnosed hemosiderosis, bacteremia, or fungemia. The risks and benefits of ferumoxytol in such patients compared to cyclic gadolinium-based contrast agents or iodine-based contrast agents must be carefully weighed.

The patient population anticipated to benefit from 3D modeling includes those with reported difficulty in access cannulation despite the use of standard practice techniques. The work presented here warrants further study in the controlled environment of a randomized clinical trial to determine if 3D dialysis access modeling of fistulas and/or synthetic grafts can improve patient care in conjunction with cost savings.

## Supplementary Material

Supplementary File 1 (PDF) Figure S1.

Supplementary File 2 (MOV) Movie S1.

Supplementary File 3 (MOV) Movie S2.

Supplementary File 4 (MOV) Movie S3.

Supplementary File 5 (MOV) Movie S4.

Supplementary File 6 (MOV) Movie S5.

Supplementary File 7 (MOV) Movie S6.

Supplementary File 8 (MOV) Movie S7.

Supplementary File 9 (MOV) Movie S8.

## References

[1] Brown R.S. (2020). Barriers to optimal vascular access for hemodialysis. Semin Dial.

[2] Monroy-Cuadros M., Yilmaz S., Salazar-Bañuelos A., Doig C. (2010). Risk factors associated with patency loss of hemodialysis vascular access within 6 months. Clin J Am Soc Nephrol.

[3] Wagner J.K., Fish L., Weisbord S.D., Yuo T.H. (2020). Hemodialysis access cost comparisons among incident tunneled catheter patients. J Vasc Access.

[4] Murphy E.A., Ross R.A., Jones R.G. (2017). Imaging in vascular access. Cardiovasc Eng Technol.

[5] Food and Drug Administration https://fda.gov/drugs/drug-safety-and-availability/fda-drug-safety-communication-fda-warns-gadolinium-based-contrast-agents-gbcas-are-retained-body.

[6] Weinreb J.C., Rodby R.A., Yee J. (2021). Use of intravenous gadolinium-based contrast media in patients with kidney disease: consensus Statements from the American College of Radiology and the National Kidney Foundation. Kidney Med.

[7] Davenport M.S., Perazella M.A., Yee J. (2020). Use of intravenous iodinated contrast media in patients with kidney disease: consensus statements from the American College of Radiology and the National Kidney Foundation. Radiology.

[8] Lu M., Cohen M.H., Rieves D., Pazdur R. (2010). FDA report: Ferumoxytol for intravenous iron therapy in adult patients with chronic kidney disease. Am J Hematol.

[9] Chin M.S., Steigner M., Yin W., Kwong R.Y., Siedlecki A.M. (2018). Intraluminal assessment of coronary arteries with ferumoxytol-enhanced magnetic resonance angiography. JACC Cardiovasc Imaging.

[10] Cheng G.Z., Estepar R.S.J., Folch E., Onieva J., Gangadharan S., Majid A. (2016). Three-dimensional printing and 3D slicer: powerful tools in understanding and treating structural lung disease. Chest.

[11] Addendum A and Addendum B Updates CMS.gov. https://www.cms.gov/Medicare/Medicare-Fee-for-Service-Payment/HospitalOutpatientPPS/Addendum-A-and-Addendum-B-Updates.

[12] Sigovan M., Gasper W., Alley H.F., Owens C.D., Saloner D. (2012). USPIO-enhanced MR angiography of arteriovenous fistulas in patients with renal failure. Radiology.

