# Supplementary figures and images for: Potential Role of 3-Dimensional Printed Vascular Models in Maintenance Hemodialysis Care
Source: Kidney Med. 2021 Sep 24;3(6):1095–8. doi: 10.1016/j.xkme.2021.07.006 (PMC8664745; doi:10.1016/j.xkme.2021.07.006)

Figure S1

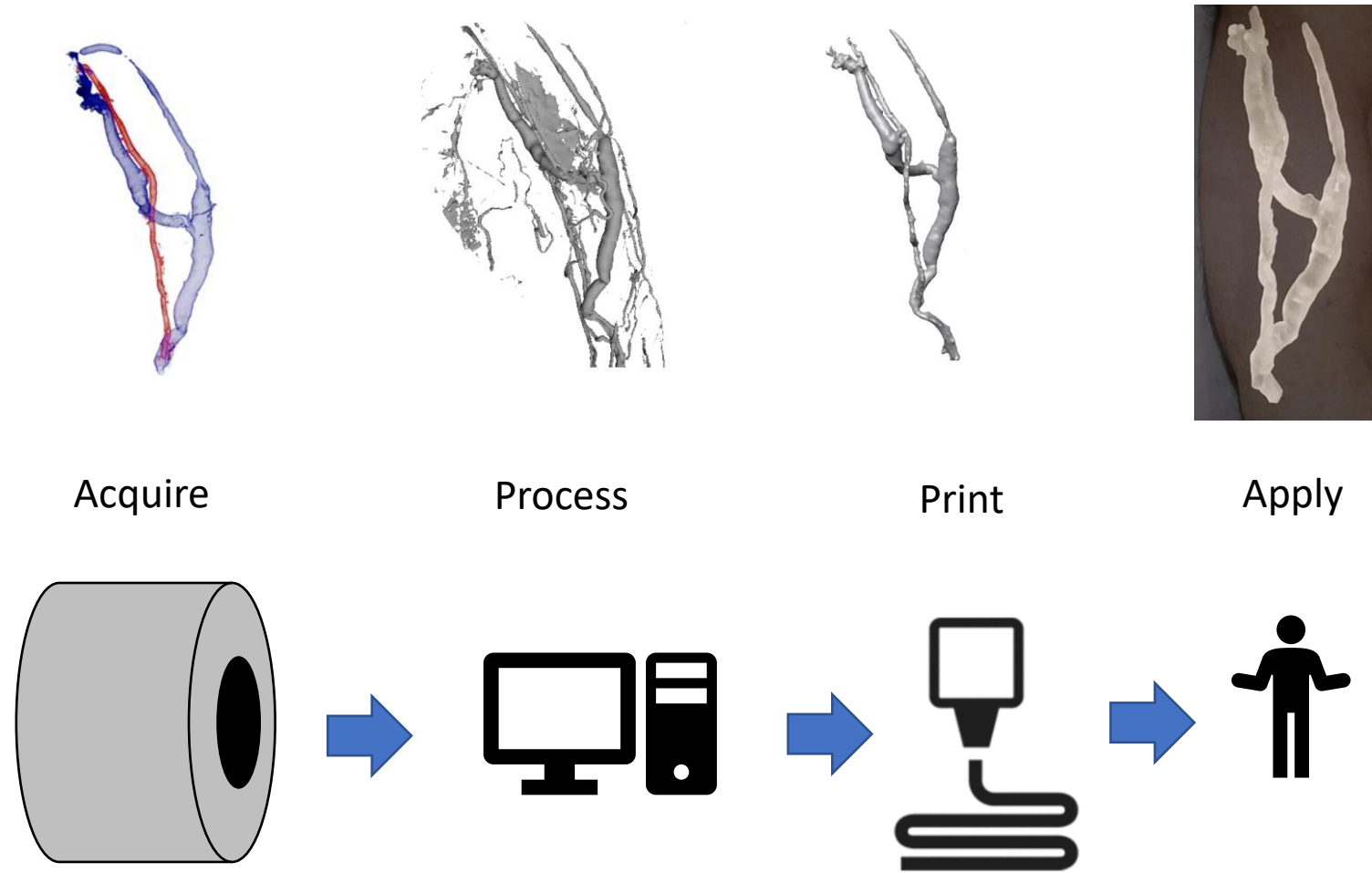

Supplement: Supplementary File 9 (MOV) — Movie S8. [file mmc9.pdf]
